# Screening and Early Identification of Spinal Deformities and Posture in 311 Children: Results from 16 Districts in Slovakia

**DOI:** 10.1155/2019/4758386

**Published:** 2019-03-17

**Authors:** Róbert Rusnák, Marina Kolarová, Ivana Aštaryová, Peter Kutiš

**Affiliations:** ^1^Central Military Hospital in Ružomberok, Neurosurgery Clinic, Slovakia; ^2^Catholic University in Ružomberok, Faculty of Health, Department of Physiotherapy, Slovakia

## Abstract

**Objective:**

In our study, we wanted to identify the number of existing deformities of the spine and posture in primary schoolers.

**Methods:**

The sample consisted of 311 healthy pupils aged 6-7. We used Klein, Thomas, and Mayer method to evaluate the posture. The spine curvature was evaluated by plumb line. Muscle imbalance was evaluated by standardized manual tests by Professor Janda. The results were evaluated by the basic population abundance and the use of the ANOVA program. We determined the level of statistical significance at p = 0.05.

**Results:**

The statistically significant occurrence of poor posture was found. Poor posture occurred in more than 50% of the pupils studied. Spine deformities in the sagittal plane have exceeded 30% (C = 37.94212%; Th = 32.15434%; L = 30.22508%). In the frontal plane deformities were present in 13.18328% of pupils. Spinae and postural disorders were accompanied by the muscle imbalance (muscle stiffness and weakness).

**Conclusion:**

Screening is a well-founded technique for the early detection of spinae and posture disorders. Based on the results of screening, professionals can take preventive measures. As in our research prevalence of spine deformities and poor posture in children was high, we recommend regular screening in clinical practice.

## 1. Introduction

Diseases of the musculoskeletal apparatus are some of the most common diseases in childhood. They are considered to be the oldest known human diseases. The first written references to their occurrence and treatment come from an old Indian religious mythological book from 3500-1800 BC. [1].

Currently, diseases of musculoskeletal system are the most frequent diagnosis because of which children visit the doctor. In Australia children less than 18 years old are commonly managed in primary care, at a rate of 5.8 (95% CI: 5.6-6.1) per 100 encounters because of musculoskeletal problems. This can be extrapolated to an estimate of 880,000 musculoskeletal problems in children and adolescents managed per year in Australia [2]. The American Association of Orthopaedic Surgeons describes the annual occurrence of abnormalities in the musculoskeletal system in 9.6 million children under the age of 19 [3]. Brzek et al. [4] report the incidence of musculoskeletal disorders in Poland ranging from 30 to 69%.

Deformities of the musculoskeletal apparatus, but especially spine and posture, are a serious problem of children [5]. Particularly scoliosis represents the most frequent diagnosis for children visiting the rehabilitation department [6]. According to American Department of Education [7], The National Scoliosis Research Society estimates that six million Americans have scoliosis, a lateral or side-to-side curvature of the spine. In the United Kingdom the prevalence of adolescent idiopathic scoliosis is estimated to be 2% to 3% of children between 10 and 16 years of age, using a definition of over 10° spine curvature. Larger curves present at a lower frequency and it is estimated that 40-degree curves make up 0.1% of the total AIS population, whereas the frequency of curves between 20 and 30 degrees is approximately 0.3 to 0.5%. A recent Japanese cross-sectional study assessed the prevalence of curvature over 10° in an 11- to 12-year-old age group and a 13- to 14-year-old age group [8]. Idiopathic scoliosis is the most common paediatric musculoskeletal disorder that causes a three-dimensional deformity of the spine. Early detection of this progressive aliment is essential [9].

The occurrence of spinal deformities in children has alarming proportions [10]. It is partly caused by the current lifestyle of children, families, and entire communities, which is characterized by hypokinesia and long-term overloading of the locomotory system in a postural disadvantageous position as sitting. The lack of movement and long-term sitting contribute to the increased occurrence of spinal deformities and poor posture in children [11–13]. Increased occurrence of poor posture and spinal deformities in children is indicated by experts from several countries. Professor Janda considers them a pandemic of modern times [14]. The amount of time spent sitting increasing once children start attending school and the natural movement of the children decreasing create ideal conditions for the formation of poor posture and spinal deformities. The incidence of these diagnoses was monitored in 16 districts of Slovakia.

## 2. Materials and Methods

Assessment of the spinae and posture was carried out in the school year 2016/2017. The sample consisted of 311 pupils aged 6 and 7. The monitored pupils were free of neurological, orthopaedic, vestibular, and other congenital or acquired disorders. There were pupils without diagnosed disorders of the musculoskeletal system. Pupils were examined with the consent of their legal guardians. The examination was carried out within the project “Healthy Backbone" under the auspices of the National Sports Centre and the Ministry of Education, Science, Research and Sports of the Slovak Republic. For the posture analysis, we chose the aspexy. The pupils were barefoot and in underwear. The examination was carried out in distance of 1 m.

To evaluate the posture, we chose the methodology according to Klein and Thomas and Mayer [15]. The methodology evaluates 5 sections of the body: (I) head and body position, (II) chest shape, (III) shape of the abdomen and bowl inclination, (IV) total curvature of the spine in the sagittal and frontal plane, and (V) height of the shoulders and position of the shoulders. The positioning of the segments is expressed by a numerical value of 1 to 4. The physiological position of the body segment is expressed by 1, a good posture is expressed by 2, a faint posture is indicated by 3, and a value of 4 represents a poor posture. The points for each segment are counted and the overall score is obtained. Based on the overall score, the subjects are categorized in 4 postural categories. The lower the overall score, the better the child's posture, and vice versa.

Groups are as follows:

- Perfect posture 5 points (postural category A)
- Good, almost perfect posture 6–10 points (postural category B)
- Faint posture 11–15 points (postural category C)
- Poor posture 16–20 points (postural category D)

The spine curvature was evaluated by a plumb line. We observed the presence of deformities in the sagittal plane (hyperlordosis and hyperkinesis) and in the frontal plane (scoliotic posture). We examined the plumb line from the header. When evaluating the curvature of the spine in the sagittal plane, the plumb line from the head should touch the kyphosis, pass through the intergluteal line, and fall between the heels, the size of the lordosis can be 2.5-3 cm, and the size of the cervical lordosis could be within 2-2.5 cm [15]. Larger or smaller variations were evaluated as pathology. In the sagittal plane we mainly observed the occurrence of deformities in the thoracic part of the spine. The thoracic spine, according to the literature, is the most common form of deformities [16–18].

If the plumb line led from the head, passed through the centre through an intangible line, and fell to the heel, we excluded the scoliotic curvature. The deviation of the curve to the right or to the left of the plumb line confirmed the scoliotic posture [15].

Spinal curvature disorders and postural disorders are accompanied by a disorder of the soft tissue: muscular and ligamentous apparatus. The condition of soft tissue was monitored by standardized manual tests, according to Professor Janda.

We tested weakening of the muscles, shortening (stiffness) of the muscles, and hypermobility. We choose postural muscles that, according to Janda [19], directly affect posture and spine curvature. Muscle evaluation was performed under standardized conditions while maintaining all testing policies and rules.

Muscle strength was evaluated for muscles that are subject to weakening: cervical flexors, m. rectus abdominis, m. obliques abdominis externus et internus, middle and lower blade fixators (mm. rhomboidei, m. trapezius middle and lower part), and mm. glutei maximi. Determination of muscle strength was assessed by six basic grades, which express the percentage of the maximum muscle strength:

- Grade 0-0%, no active movement present
- Grade 1-10%, only muscle contraction present
- Grade 2-25%, muscle contracted by a quarter of normal muscle strength
- Grade 3-50%, muscle contracted by a half of the normal muscle strength
- Grade 4-75%, muscle contracted by three quarters of normal muscle strength
- Grade 5-100%, representing normal, physiologic muscle strength [19, 20]

Muscle stiffness was tested for muscles that are subject to shortening. We tested the following muscles: m. trapezius upper fibres, mm. erectors of the spinae, m. pectoralis major, m. quadratus lumborum, and m. iliopsoas. When testing shortened muscles, we distinguished three degrees of abbreviation:

- Grade 0: the muscle was not shortened; the joint had physiologic movement
- Grade 1: the muscle was shortened; the joint movement was slightly limited
- Grade 2: the muscle was significantly shortened; the joint movement was limited [19, 20]

If we found higher movement in joint, that is physiologic, we classified it as hypermobility.

The results of the examinations were processed in MS EXCEL, 2013. We used descriptive statistics methods for results analysis. In order to verify the assumptions, we used a base share count test and ANOVA program. We determined the level of statistical significance at p = 0.05.

In order to verify the first assumption of correct posture, we used mathematical hypotheses formulated in the form of a zero and alternative hypothesis. We assumed the correct body posture in at least then 50% of primary school pupils. The accuracy of the assumptions has been verified by using a base share count test. We tested the hypothesis of the consistency of the base set *π* and the constant *π*_0_.

The zero hypothesis: H_0_: *π* = *π*_0._

The alternative hypothesis for a two-sided test: *π* ≠ *π*_0._

In the case of a one-sided test, we have formulated an alternative hypothesis: *π* < *π*_0,_ or *π* > *π*_0._

The range of the set n = 311 was high enough and the distribution of the sample could be approximated by the normal distribution $N=\left(\pi ,\sqrt{\left(\pi .\left(1-\pi \right)\right)/n}\right)$ and use the test statistic

(1) $$\begin{array}{c} z=\frac{\pi -\pi_{0}}{\sqrt{\left(\pi .\left(1-\pi \right)\right)/n}} \end{array}$$

for division N (0,1). We compared the test characteristic with the critical value of z. If |*z*| < *z*_∝_, we recommend accepting a zero hypothesis. Otherwise, we recommend accepting the alternative hypothesis.

To verify second assumption, we used the ANOVA program. We have formulated the basic hypothesis:

(2) $$\begin{array}{c} H_{0}\text{:}\;\;m_{1}=m_{2}=m_{3} \end{array}$$

H_1_: in at least one pair of mean values there is not equality.

Let there be *m*_*i*_ ≠ *m*_*j*_, i≠j, i, j=1,2,3.

We determined the level of statistical significance at p = 0.05.

## 3. Results 

The results of the assessment of the posture and the distribution of the pupils in the postural categories are presented in Table 1. The total number of pupils studied was three hundred and eleven (n = 311). Column A presents the number of children whose posture has been rated as perfect. Overall score to classify to this postural category, according to Klein, Thomas and Mayer was 5 points. Column B presents the number of children whose posture has been rated as good, almost perfect posture. Overall score to classify to this postural category, according to Klein, Thomas, and Mayer, was 6–10 points. Column C presents the number of children whose posture has been rated as faint posture. Overall score to classify to this postural category, according to Klein, Thomas, and Mayer, was 11–15 points. And column D presents the number of children whose posture has been rated as poor posture. Overall score to classify to this postural category, according to Klein, Thomas, and Mayer, was 16–20 points. Correct posture was the sum of the children in the A + B category and it is presented in column A + B. The incorrect posture corresponded to the sum of the children of the C + D group and it is presented in column C + D.

Out of 311 examined pupils, 53 pupils had perfect posture and were classified to postural category A. Good, almost perfect posture was assessed in 118 pupils and they were classified to postural category B. Faint posture was evaluated in 99 pupils and they were classified to postural category C. Poor posture was present in 41 pupils and these pupils were classified to postural category D. Correct posture was evaluated in 171 children (column A + B) and incorrect posture was evaluated in 140 children (column C + D).

Quantity for correct posture: n_s_= 171; *π*=171/311=0,549839; q= 1-*π* = 0,450161; constant *π*_0_=0,5.

Test characteristics: $z_{stat}=\left(0,549839-0,5\right)/\sqrt{\left(0,549839*0,450161\right)/311}=$1,766647, z0,05= 1,644854.

The calculated value of “z” is higher than the critical value, so we recommend accepting an alternative, one-sided hypothesis. A statistically significant occurrence of incorrect posture in children was found in the study. It occurred in more than 50% of the pupils studied.

Additional assumption was that the most critical part of the spine that is most affected by the deformities is the thoracic spine in the sagittal plane. The occurrence of all pathological curvature of the spine is shown in Table 2.

In the sample the pathological curvature of the spine in the sagittal plane was found as follows: curvature defects in the cervical spine (C) were found in 118 pupils (37.9%), in the thoracic spine (Th) we found curvature disorders in 100 pupils (32.2%), and we evaluated pathological curvature in lumbar spinae (L) in 94 pupils (30.2%). Deformities in the frontal plane (scoliotic curvature) were found in 41 pupils (13.2%).

After the assessment of the thoracic spine in the sagittal plane, we calculated following parameters, as shown in Tables 3 and 4.

In the first test (Table 3), where we tested only the thoracic (Th) curvature at a level of significance of 0.05, thoracic (Th) curvature proved as being statistically significant (Table 3, values z_stat_ > z_krit_). Is this curvature significantly larger than the other spine curvatures?

To verify this assumption, we created Table 4 and we used ANOVA program. In Table 4, we present the occurrence of spine curvature defect in children and the spine curve defects from the most affected section to the least affected segment of the spine.

Deformities of the thoracic spine (Th) in the sagittal plane are the second most frequently occurring disorder compared to curvature defects in the cervical spine (C) and lumbar (L) spine. Are the results comparable or is there a significant deviation between them? We verified the results using the ANOVA program. Results are showed in Tables 5 and 6.

Based on the critical F value (F_crit_=3.204), statistical F value (F_stat_=3.204), and value P (P= 0.54) presented in Table 6, there was no significant statistical difference between the groups. Based on our calculations, no significant statistical difference was found between deformities of thoracic and cervical and lumbar spine. The test results confirmed that the curvature of the spine in the thoracic area is not the most critical segment that is subject to deformity. The assumption has not been confirmed.

Spinal curvature disorders and postural disorders are accompanied by a disorder of the soft tissue: muscular and ligamentous apparatus. The condition of soft tissue is presented in Tables 7 and 8. In Table 7 we present muscle imbalance (shortening and weak muscles). Left part of Table 7 presents shortening muscles and right part presents weak muscles. In the table we present the average values of shortening and weakening of the muscles, the standard deviation, the most frequently occurring shortening, respectively, weakness muscle values (MODE), median values, and the minimum (MIN.) and maximum (MAX.) measured values of muscle tests.

Muscle weakening was followed for muscles: mm. flexors cervicis, mm. scapula fixators (mm. rhomboidei, m. trapezius medial and caudal part), mm. abdominis (m. rectus abdominis, mm. obliquues externi et interni), and mm. glutei maximi.

Blade fixators and abdominal muscles were assessed by grade 3 of the muscle test (in the table MIN.). This was the lower value of the muscle weakness that we measured. For the other muscle we evaluated minimum muscle strength by grade 4 (in the table MIN.). We did not evaluate any muscle with values from 2 to 0.

In our research group the most common weakened muscles were shoulder blade fixators, where the mean value of weakness reached 4,051±0,559. Median value for shoulder blades fixators reached 4, which indicates that half of the pupils under the study had physiological strength of the monitored blades muscles. Weakness of the mm. abdominis (average weakness value 4,408±0,711) took the second place; then, it is followed by cervical flexors (average weakness value 4,576±0,494) and mm. glutei maximi (average weakness value 4,698±0,459). In these muscles median reached value 5, which meant that at least half of the pupils under the study had physiologic muscle strength.

We evaluated shortening (stiffness) for the muscles: m. trapezius (cranial part), mm. iliopsoas, mm. pectoralis majors, mm. erectors of the spinae, and mm. quadratus lumborum. In Table 7 we present values for bilateral measurements.

With the value 2 (in table MAX.), which means significant muscle shortening, we only evaluated m. iliopsoas. This was the only muscle evaluated with all muscle test grades from 2 to 0. All the other muscles were evaluated with values from 1 to 0, which meant that we evaluated just slight muscle shortening or physiologic muscle length.

The most common shortened muscles were mm. pectoralis majors. The average shortening in these muscles was 0,804±0,397. The median value for mm. pectoralis majors was 1, which meant that half of the pupils under the study had physiological length of the monitored pectoralis muscles. Shortening of the mm. iliopsoas (average shortening value 0,588±0,711) took the second place; then it is followed by mm. trapezius cranial part (average shortening value 0,379±0,485) and mm. erectors of the spinae (average shortening value 0,302±0,459). Average shortening value for m. quadratus lumborum was 0,132±0,338. Median for all the other muscles reached value 0, which meant that at least half of the pupils under the study had physiologic muscle length.

Last, but not least, we evaluated muscle hypermobility in children. We reexamined pupils whose muscles reached physiologic (normal) length. When muscle length in reexamined group exceeded physiological values, we considered it as hypermobility. Results are presented in Table 8.

According to our result, hypermobility was less presented than muscle stiffness. Hypermobility was presented in mm. trapezius upper part in 22.19% of pupils. The same result was for mm. erectors of the spinae. Mm. pectoralis majors took the third place. Hypermobility was presented in 15.43% of pupils. Then, mm. quadratus lumborum followed. Hypermobility was presented in 12.86% of students. In mm. iliopsoases hypermobility was presented in 6.11% of pupils. Backbone deformities and postural disorders were more often caused by muscle stiffness than muscle hypermobility.

## 4. Discussion

The aim of the study was to identify the spine deformities and postural disorder in the monitored children and to point out the importance of children's backbone and posture screening. Early identification of disorders, correct diagnosis, and determination of the cause of these diseases play an important role in preventing further progression [17].

Screening is process of identifying apparently healthy people who may be at increased risk of a disease or condition. They can then be offered information, further tests, and appropriate treatment to reduce their risk and/or any complications arising from the disease or condition [21]. Screening has been applied in several fields of medicine since the last century. According to Kuroki [22], the first screening program following spinal deformities was established in Minnesota in 1947. Since 1960, the program has begun to improve in the United States. Then, “school scoliosis screening program (SSSP)” arose, which has been applied in many countries of the world. MacEwen played an important role in its development. The program was officially launched in 1963 in central Minnesota by applying the “forward bending test" (Adams' forward bending test). It is used as the simplest test to diagnose scoliosis to date. School scoliosis screening program has spread from America to the following countries: in 1970 to Canada, Great Britain, Australia, and Norway; in 1977 the SSSP was applied in Sweden, Greece, Poland, Dublin, and Hong Kong. Countries such as China, Bulgaria, Spain, Israel, Singapore, Italy, Turkey, or Malaysia were volunteered. In Japan, spinal screening is governed by the law [22].

Regular screening of spine in children is recommended today. The American Academy of Orthopaedic Surgeons proposes regular screening of children aged 11 while the American Academy of Paediatricians has proposed regular screening of the backbone of school-age children from 10 years of age [13].

Currently, in our country, backbone and posture screening as part of preventive health care for children is not being implemented. Children are recommended rehabilitation or sent to children's orthopaedics only when there are more and more visible deformities that parents or pedagogues notice. Occasionally, the deformity can be detected accidentally when the child visits a doctor for quite another reason (trauma, back pain, etc.). Then children are examined at their parents' request. For this reason, in many cases the onsets of deformities are not detected in time. In our follow-up file, the occurrence of spine deformities and poor posture was high. We have deliberately chosen a research sample consisting of healthy population of pupils who, at the time of the examination, were not diagnosed with any musculoskeletal disorder. In our research sample there were not children with existence of any unusual skeletal deformity such as operated club foot, polydactyly, or cleft lip or palate. Despite this, the musculoskeletal system of most of the children under study was disturbed. We observed the occurrence of spinal deformities in the sagittal and the frontal plane. In the sagittal plane, the incidence of spine deformities exceeded 30%. Spine deformities in the sagittal plane were represented as follows: spine deformities in the cervical spine of 118 pupils (37.9%), thoracic 100 pupils (32.2%), and lumbar 95 pupils (30.2%). Pupils either suffered from only one spine defect or had curvature defects of two to three sections. Deformities in the frontal plane, scoliotic curvature, were found in 13.2% of pupils.

Based on the literature where kyphosis and hyperkyphosis are described as the most common deformity of the spine in childhood [16–18] and that thoracic kyphosis is dominant at the age of 6-7 [23], we predicted that the most critical part of the spine most affected by the deformities will be the thoracic spine. This assumption has not been confirmed. We compared all three spinal sections in the sagittal plane and we evaluated them with the ANOVA program. There was no significant statistical difference. The thoracic curvature was not the most critical segment that underwent deformity. We can agree with the claims of Jandrić [24], who states that all three spinal sections are subject to deformities. Pathological findings in all areas of the spine are described by several experts dealing with this issue. Brianzi et al. [25] monitored 201 children of Sao Paulo and recorded the following prevalence of postural deformities and asymmetry in children. They found cervical anteflexia in 40.30% of children, hyperkyphosis in 43.78% of children, hyperextension in lumbar part in 49.75% of children, scoliosis type “C” in 41.3% of children, and scoliosis “S” type in 9.95% of children. Penha et al. [26] (2008) followed the body posture in 191 children aged 7 to 10 years. The confidence level for tested segments was 0.75. Values below 0.75 were considered pathological. In their study the most critical were the curvatures of the cervical spine, which reached level of 0.43. Hyperlordosis of cervical backbone occurred in 50.64% of children. In the thoracic spine pathology reached level of 0.68 and just above the borderline was lumbar spine and it reached 0.78. Nikšić et al. [12] monitored 1105 children aged 5 to 12 and described the occurrence of first-degree spine deformities: kyphosis, scoliosis, and lordosis in 37.5% of pupils, and the occurrence of second-degree spine deformities (combination of deviations or individual second-degree disorders) in 16% of pupils. In their observed sample spinal deformity in the frontal plane occurred in 21.8% of boys and 19.4% of girls. Stanojković et al. [27] reported that, of the 236 primary school pupils they observed, kyphosis was observed in 61.7% of boys and 76.2% of girls, and lumbar hyperlordosis was observed in 3.7% of pupils studied and scoliosis was observed in 64.5% of students. Femić et al. [16] observed the incidence of kyphosis and scoliosis in children in the second year of elementary school. The thoracic spine deformities were reported in 78.26% of cases, while scoliosis was reported in 17.39% of children.

As we noted in the text, in America the screening of spine deformities is recommended in 10 to 11 years of life. However, our study showed that spinal deformities may occur in children much earlier, that is, when children are admitted to school (6-7 years). Similarly, to our study, Jahle and Kuhins [28] describe the occurrence of spinal deformities and postural disorders in children in the first year of primary school. These authors followed 367 children in Switzerland and found that 55% of children in the first year had spinal and posture disorders, while in the third year their number increased to 66.5%. The disorders were significantly related to the cervical and lumbar spine. Spinae and postural disorders in age of 7 are also described by Brianezi et al. [25] and Lafond et al. [29].

Deformities of the spine are closely related to incorrect posture, and these two issues are often combined. In our study we observed the statistically significant occurrence of incorrect posture in children, which allows us to assume that this trend is of a general nature. Thus, the general incorrect posture occurred in more than 50% of the pupils studied. Of the total number of 311 primary school pupils studied, 53 pupils (17%) had excellent body posture, 118 had a good posture (37.94%) and were included in postural category B, in postural category C, flimsy posture, the number of children was 31.83% (99 children), and in postural category D, bad posture, the number exceeded 13% (41 children). Similar results were published from other countries, as well. In the Czech Republic, Kratěnová et al. [30] monitored posture in 3520 children in 10 cities and they recorded incorrect posture of the body in 38% of monitored pupils. Similar incidence of the body and the musculoskeletal disorders (40.86%) was observed in smaller study from Montenegro with children enrolled in elementary school [29]. In Bulgaria, Mitova reports high occurrence of nonphysiological behavioural disorder. Out of 2129 children she observed, 58.85% of the monitored population has postural disorders [10]. Lafond et al. [29] evaluated children's posture in Canada. They evaluated the deviations of the posture in the sagittal plane. Their results indicate that the children aged 4–12 years have pathologic posture in sagittal plane and it is characterized by the forward head position, forward shoulders position, and pelvis and knees forward position. In Israel, 62.4% of the children surveyed had body posture issues [31]. Chaves, Oliveira, and Damázio [32] examined 117 schoolchildren in Brazil and stated that high incidence of postural changes was observed in the studied population. About 56% (n = 14) presented some type of head alteration and the other 44% (n = 11) presented no head position changes. Among the postural alterations evaluated in the head it was observed that 12% presented head protrusion and 44% presented head tilt to the right or left. 64% presented a shoulder elevation and 24% had a shoulder protrusion. Other postural alterations were observed in the studied population, and 67.27% (n = 74) had pelvic alterations, such as anteversion and pelvic retroversion. Other postural alterations were found, such as cervical hyperlordosis, thoracic hyperkinesis, lumbar hyperlordosis, and thoracolumbar scoliosis. In the knee, changes were identified as valgus knee, varus knee, and recurvatum knee.

Backbone and postural disorders in our research group were caused by the muscle unbalance, muscle weakness and stiffness.

The prevalence of postural insufficiencies in children is high [33]. Studies show that there are genetic, ergonomic, and lifestyle factors that may trigger these postural changes [32]. Studies also show that schoolchildren with heavy backpack loads show postural changes [34, 35] as well as obesity in children [36].

In our article we tried to approach the issue of spinal deformities and posture disorders in school-age children and point out the importance of its screening. However, we have encountered certain limitations in our research.

For limitation of our research we consider our ability to diagnose spinae and posture in children. Diagnosis of the spine and of posture was limited, in our case, to visual analysis. At present time, there are several modern computerized diagnostic methods that are applied in clinical practice. Unfortunately, access to computer assessment of spine and posture in our workplaces is currently not available and therefore we have been limited to visual diagnostics. The size of the deformities cannot be seen from our examination and it cannot be judged whether these deformities are functional or structural. We recommend completing an examination by photography and computational analysis in specialized clinics. The limitation of our research was also the subjectivity of the examination technique compared to the computerized techniques. Although the methodology of Klein, Thomas, and Mayer that we used in spinae and posture analysis accurately describes the numerical values of the position of body segments and spinal segments, the physiotherapist's professional experience (years of practice, number of examined children, etc.) plays a role in analysing and in the postural evaluation. To rule out this subjective factor, the posture of school children was always analysed by the same physiotherapist. Our research group was small. It consisted of 311 school children from 16 districts in Slovakia. We consider it as another limitation of our study. Screening and evaluating of spine and posture should be extended to a larger population of schoolchildren.

The Klein, Thomas, and Mayer methodology is available, a simple, unexpansive, and reliable diagnostic method, just like the Adams forward bending test mentioned above. Therefore, any physiotherapist, podiatrist, orthopaedist, or physician doctor may use it in their practice to monitor the incidence of spine deformities and postural disorders and propose timely preventive and therapeutic treatment. We consider this as an advantage of the diagnostic's methodology used by us in our research.

On the basis of facts which we have gained in our research, we would suggest application of screening of schoolchildren's spine and posture in clinical practice. We suggest regular backbone and posture screening in children at the beginning of compulsory school attendance. In this period of child's life, the musculoskeletal system is the most overloaded by carrying a school bag, long-term sitting, and a general change of lifestyle. It increases in children in the second year of elementary school (age of 7-8). Children in this age represent a critical group for the development of spinal deformities and postural disorders [18]. According to Demeš-Drljan, Mikov [18], this is the reason why evaluation of spine curvature and posture should be commenced when children start attending school and repeated in critical ages. We also suggest regular screening of school children's spine starting in the first grade in primary school and repeating it in critical ages (second grade of primary school and in a period of intense growth, age of 10 to 15). We also recommend monitoring the impact of the long-term sitting and the effect of wearing a school bag and monitoring the effect of movement activities of children on their spine and posture.

As our study was aimed at basic school screening program and apparently healthy population, we used only three diagnostics methods to evaluate backbone and posture in children. Although we found serious postural deformities (postural categories C and D) and high frequency of spinal deformities in children, we recommended to their parents visiting rehabilitation department for complex backbone and postural diagnostic. Complex backbone and posture diagnostics in Slovakia consist of special kinesiology examination such as examination of backbone movement, X-ray examination, exploring the skin for any unusual skin stigmata, exploring the muscle spasm or pain, exploring the muscle trigger or tender points, hyperalgic zones, following of family history of skeletal deformities, and following of occurrence of congenital or acquired developmental errors. According to this complex analysis doctors and physiotherapists can create individual treatment program for children and limit pathologic spinae progression.

## 5. Conclusion

Results from our research and from several countries around the world highlight the unfavourable situation in children's spine and posture. This needs to be addressed. Therefore, we consider screening of the spine and posture in children to be justified. Spinal and postural school screening program offers useful information about health status of children. Evaluation of the spine deformities and postural disorders gives the opportunity to identify the incidence and prevalence of these diseases. At the same time, it enables timely and adequate intervention and development of treatment and preventive programs for children aimed at halting the progression of these musculoskeletal disorders.

Based on the results of our study and recommendations of the UK National Screening Committee [21], the children we investigated in this research are included in the “Healthy backbone" preventive program, which means that every year they will be reexamined. All children practice daily health exercises created by our specialists. Teachers of the examined children attended a specialized seminar created by our specialist and they apply these healthy exercises in schools. Parents of children who have been diagnosed as having postural or backbone disorders have been provided with information on how to prevent further progression of deformities or have been sent for a specialist treatment. Currently we try to excite the project in all the districts of Slovakia and after 4 years of activity evaluate its success.

As in modern medicine and thus in rehabilitation, emphasis is placed on the prevention of civilization diseases, where spinal deformities and poor posture belong, and the screening of spine and posture of children in school age should be popularized and applied in common practice.

## Figures and Tables

**Table 1:** Pupils based on posture assessment.

*A *	*B*	*C*	*D*	*n*	*A+B*	*C+D*
53	118	99	41	311	171	140

**Table 2:** Spinal curvature assessment (n=311).

	Sagittal plane			Frontal plane
	*C*	*Th*	*L*	*Scoliotic curvature*
No of pupils with deformities	118	100	94	41
Pathology occurrence in %	37,94212	32,15434	30,22508	13,18328

**Table 3:** The pathologic curvature of thoracic spinae in sagittal plane.

*n*_*Th*_	*π*	*1-π*	*π. (1-π)*	*z*_*stat*_	*z*_*crit*_
100	0,321543408	0,678457	0,218153	8,364851	1,644854

**Table 4:** Order of the occurrence of spine curvature deformities in sagittal plane.

Spine section	No of pupils with deformities	Deformities in %	Order of deformities
C	118	37,94212	1
Th	**100**	**32,15434**	**2**
L	94	30,22508	3

**Table 5:** Sagittal level deformities (n=311).

Groups	Count	Sum	Average	Variance
C	16	118	7,375	15,05
Th	**16**	**100**	**6,25**	**14,86666667**
L	16	94	5,875	17,05

**Table 6:** Source of variation (n=311).

Source of Variation	SS	df	MS	*F*_stat_	P-value	*F*_crit_
Between Groups	19,5	2	9,75	**0,623**	**0,54**	**3,204**
Within Groups	704,5	45	15,66			
Total	724	47				

**Table 7:** Shortening and weak muscle test.

	pectoralis majors bilat.	*mm. Iliopsoases* *bilat.*	trapezius cranial parts bilat.	erectors spinae bilat.	quadratus lumborum unilateral	scapula fixators bilat.	musculi abdominis bilat.	flexors cervicis bilat.	glutei maximi bilat.
AVERAGE	**0,804**	0,588	0,379	0,302	0,132	**4,051**	4,408	4,576	4,698
MODE	**1**	0	0	0	0	**4**	5	5	5
MEDIAN	**1**	0	0	0	0	**4**	5	5	5
STANDR	0,397	0,711	0,485	0,302	0,338	0,559	0,711	0,494	0,459
DEVIATION									
MIN.	0	0	0	0	0	**3**	**3**	**4**	**4**
MAX.	**1**	**2**	**1**	**1**	**1**	5	5	5	5

**Table 8:** Comparison of hypermobile, stiffness, and physiologic muscle length (n=311).

	mm. trapezius		mm. erectors spinae		mm. pectorals majors		mm. quadratus lumborum		mm. iliopsoas	
	No	%	No	%	No	%	No	%	No	%
hypermobile	69	22.19	69	22,.9	48	15.43	40	12.86	19	6.11
stiffness	117	37.62	117	37.62	249	80.07	40	12.86	141	45.34
normal	125	40.19	125	40.19	14	4.5	231	74.28	151	48.55

## Data Availability

The anonym examination results data used to support the findings of this study are available from the corresponding author upon request.
